# Reactivating Ovarian Function through Autologous Platelet-Rich Plasma Intraovarian Infusion: Pilot Data on Premature Ovarian Insufficiency, Perimenopausal, Menopausal, and Poor Responder Women

**DOI:** 10.3390/jcm9061809

**Published:** 2020-06-10

**Authors:** Konstantinos Sfakianoudis, Mara Simopoulou, Sokratis Grigoriadis, Agni Pantou, Petroula Tsioulou, Evangelos Maziotis, Anna Rapani, Polina Giannelou, Nikolaos Nitsos, Georgia Kokkali, Michael Koutsilieris, Konstantinos Pantos

**Affiliations:** 1 Centre for Human Reproduction, Genesis Athens Clinic, 14–16, Papanikoli, 15232 Athens, Greece; sfakianosc@yahoo.gr (K.S.); agnipantos@gmail.com (A.P.); lina.giannelou@gmail.com (P.G.); nitsos@otenet.gr (N.N.); georgiakokkali@gmail.com (G.K.); info@pantos.gr (K.P.); 2 Department of Physiology, Medical School, National and Kapodistrian University of Athens, 75, Mikras Asias, 11527 Athens, Greece; sokratis-grigoriadis@hotmail.com (S.G.); petroulatsi@yahoo.gr (P.T.); vagmaziotis@gmail.com (E.M.); rapanianna@gmail.com (A.R.); mkoutsil@med.uoa.gr (M.K.)

**Keywords:** platelet-rich plasma, intraovarian infusion, ovarian rejuvenation, ovarian reserve, poor responders, perimenopause, menopause, premature ovarian insufficiency

## Abstract

Intraovarian platelet-rich plasma (PRP) infusion was recently introduced in the context of addressing ovarian insufficiency. Reporting on its effectiveness prior to adopting in clinical routine practice is imperative. This study aims to provide pilot data regarding PRP application for ovarian rejuvenation. Four pilot studies were conducted on poor ovarian response (POR), premature ovarian insufficiency (POI), perimenopause, and menopause, respectively. Each pilot study reports on thirty patients, 120 participants were recruited in total. All participants provided written informed consent prior to treatment. Primary outcome measures for the POR pilot study were levels of anti-müllerian hormone (AMH), antral follicle count (AFC) and oocyte yield. For the POI, perimenopausal and menopausal pilot studies primary outcome measures were restoration of menstrual cycle, and Follicle Stimulating Hormone (FSH) levels. A significant improvement on the hormonal profile and the ovarian reserve status was noted, along with improved intracytoplasmic sperm injection (ICSI) cycle performance concerning POR participants. Menstruation recovery was observed in 18 out of 30 POI patients, along with a statistically significant improvement on levels of AMH, FSH, and AFC. Similarly, 13 out of 30 menopausal women positively responded to PRP treatment. Finally, menstruation regularity, improved hormonal levels and AFC were reported for 24 out of 30 perimenopausal women. To conclude, PRP infusion appears to convey promising results in addressing ovarian insufficiency.

## 1. Introduction

Clinicians are often called to address the challenging issue of ovarian insufficiency in the context of fertility treatment [1]. The common dominator characterizing patients presenting with ovarian insufficiency is that they typically are unable to ovulate normally since they present with reduced number of stimulable primordial follicles [2]. The wide pallet of factors affecting ovarian function, ranging from advanced maternal age, to genetic, iatrogenic, or environmental factors, may add another level of complexity toward efficient management [3,4].

The principal representatives include women presenting with premature ovarian failure [5], also described as premature ovarian insufficiency (POI) [6], a condition characterized by a premature collapse of ovarian function. Women of poor ovarian response (POR) represent another major category. The definition and classification of POR patients is a topic of heated debate for the scientific community. Predominantly, patients’ categorization is based on age, along with ovarian reserve status, as it is proposed by Bologna criteria [7]. The POSEIDON (Patient-Oriented Strategies Encompassing Individualized Oocyte Number) group proposed a new stratification employing a respective developed algorithm to categorize patients based on qualitative and quantitative parameters: (a) maternal age and the expected aneuploidy rate, (b) ovarian reserve biomarker levels such as antral follicle count (AFC) and anti-müllerian hormone (AMH), and c) ovarian response data in previous cycles following ovarian stimulation protocols. This approach offers a practical endpoint to clinicians by assisting in setting a clear goal for management of the group of “low prognosis patients” in assisted reproduction treatment (ART) [8,9]. Irrespectively of the definition, POR women commonly present with scarcity of stimulable follicles resulting in low oocyte yield, and high cancellation rates following ART [8].

Otherwise healthy women of advanced maternal age, as well as perimenopausal and even menopausal women [10] similarly explore alternative options in their quest to achieve a pregnancy [4]. Additionally, diminished ovarian reserve, is coupled with poor oocyte quality, entailing a heavily compromised fertility status-irrespectively of age [11].

Current available options for managing women presenting with ovarian insufficiency are commonly in vitro fertilization (IVF) treatment, coupled with embarking on an oocyte donation program, surrogacy, or alternatively adoption [12,13,14,15,16]. Nonetheless, the pursuit of a healthy genetically linked offspring drives scientists toward investigating ovarian function restoration approaches for these patients. The respective research aims to improve the deteriorating hormonal profile along with enabling gamete production [17].

Emergence of the autologous platelet-rich plasma (PRP) intraovarian infusion reflects a break-through approach, showcasing promising results. PRP is described as plasma containing high concentrations of platelets [18]. Their granules contain a wide pallet of proteins and hormones, along with numerous growth factors, cytokines, and chemokines [19], playing an important role toward orchestration of tissue restoration and repair [20,21]. Furthermore, platelets serve as a source of proteins presenting with antimicrobial activity [18].

The application of PRP and its effectiveness was showcased in the context of regenerative medicine [22,23,24,25,26], with highly promising results. Concerning the field of reproductive medicine, PRP treatment was clinically performed with an emphasis on improving endometrial growth, and pregnancy outcome following IVF [27,28]. Moreover, endometrial PRP infusion was reported for managing chronic endometritis, resulting in live birth [29]. The radical concept of autologous intraovarian PRP ovaries infusion was pioneered [30,31], with the intent to revive ovarian function in perimenopausal women. Thenceforth, various attempts were documented entailing autologous intraovarian PRP infusion in POR patients [32,33], in menopausal and in POI women [34,35], demonstrating its promising role in infertility management. Albeit PRP has not established routine clinical status yet, it has emerged as an option in addressing ovarian insufficiency. Despite the reported encouraging results, an all-inclusive report on PRP’s clinical, cellular and molecular regenerative dynamic on the reproductive system still eludes us [36].

Application of PRP still challenges the scientific community, posing various concerns that remain to be investigated. The quest for optimal application of PRP continues.

Conduction of well-designed randomized controlled trials (RCTs) is imperative to demonstrate effectiveness of the aforementioned “add-on” treatment, prior to standard establishment into the clinical IVF practice [36]. Until data from RCTs become available, the authors sourced the present results from four pilot studies regarding PRP application for ovarian rejuvenation-outside the scope of case series or reports. The present study focuses on restoration of ovarian function following intraovarian PRP infusion, with respect to four categories of patients presenting with the pathophysiology of ovarian insufficiency. These categories include poor responders, perimenopausal women, women presenting with POI, and finally menopausal women.

## 2. Materials and Methods

The pilot studies were performed between February 2017 and January 2019 at the Centre of Human Reproduction, Genesis Athens Clinic, in order to properly investigate the efficiency of autologous PRP ovarian infusion in a wide range of pathophysiological conditions jeopardizing ovarian dynamic. The pilot studies were designed as prospective observational cohort studies. All of the studies’ participants referred to Genesis Athens Clinic individually as patients intending to address ovarian insufficiency. Based on the diagnosis reported POR, POI, perimenopause, or menopause, the participants were assigned in the respective pilot study. Thirty women fulfilling specific inclusion criteria were recruited per pilot study. Details regarding the studies’ design are summarized in Table 1.

### 2.1. General Exclusion Criteria

Women presenting with autoimmune disorders, sexually transmitted diseases, infectious diseases, tubal factor infertility/tubal obstruction, chronic inflammatory diseases, endometriosis, chronic endometritis, and endocrine disorders such as thyroid dysfunction, were excluded in general. Body Mass Index (BMI) above 30 or less than 18.5, hypothalamic-pituitary disorders, and medical history including surgeries of the reproductive tract were further considered to be general exclusion criteria. Patients presenting with anemia, thrombophilic disorders, current cancer diagnosis or a medical history of familiar cancer, were also excluded. Finally, all couples presenting with an abnormal semen analysis were excluded.

### 2.2. Participants in the Poor Ovarian Response Pilot Study

Women included in this pilot study presented with POR according to the Bologna criteria [7]. Participants were fulfilling at least two out of the three following criteria including: advanced maternal age (≥40 years old), previous POR described as previous IVF attempts resulting in fewer than three oocytes retrieved or an abnormal ovarian reserve test including an AFC of less than five follicles or AMH levels less than 1.1 ng/mL. All women reported regular length of menstrual cycle. Couples presenting with any other infertility etiology other than POR were excluded. All participants were subjected to the standard examination as described below.

At least one previous failed Intracytoplasmic Sperm Injection (ICSI)-Embryo Transfer (ET) fresh cycle was common for all participants. The Gonadotropin-Releasing Hormone (GnRH) antagonist protocol was the controlled ovarian stimulation (COS) protocol of choice in the ICSI-ET cycle prior to PRP treatment.

PRP intraovarian infusion treatment was performed at least two months following the last failed ICSI-ET cycle. In the third menstrual cycle post PRP treatment all participants received the GnRH antagonist protocol and underwent an ICSI-ET fresh cycle. The protocol included daily administration of recombinant follicle stimulating hormone (FSH) (Gonal-F, Merck, Darmstadt, Germany) at 300 IU initiated on day-two of the menstrual cycle. Adjustment of gonadotropin dosage was based on sonographically assessed follicular development. GnRH antagonist administration (Cetrotide, Merck) was initiated when the leading follicle reached 14 mm or estradiol (E2) reached 300–400 pg/mL. Final oocyte maturation was triggered with human chorionic gonadotropin (hCG) (Ovitrelle 250 mg, Merck) when leading follicles were ≥18 mm. Oocyte retrieval was performed 36 h following administration of hCG. Oocytes were denuded two hours following oocyte retrieval. The insemination method employed was ICSI similarly to the participants’ last ART cycle. Cycles were concluded with ETs including two cleavage stage embryos, when two or more embryos were available or with a single ET when one embryo was obtained. Luteal phase support was initiated immediately following oocyte retrieval, employing intravaginal (Vasclor gel 8% w/w, Verisfield, Athens, Greece) and subcutaneous progesterone administration (Prelutex 25 mg/mL, Proton Pharma A.Ε, Athens, Greece), daily, until the hCG pregnancy test was performed 14 days following ET.

### 2.3. Participants in the Premature Ovarian Insufficiency Pilot Study

Women included in this pilot study were diagnosed with POI, presenting with amenorrhea. Inclusion criteria referred to the participants’ age < 40 years, amenorrhea for at least four months, and elevated FSH levels >25 IU/L recorded on two occasions >4 weeks apart. Inclusion criteria were set according to guidelines regarding management of women with POI, established by the European Society of Human Reproduction and Embryology (ESHRE) [37].

### 2.4. Participants in Perimenopause Pilot Study

Participants included in the specific pilot study were of advanced maternal age defined as age ≥ 40 years and were presenting with cycle irregularities while fulfilling at least one of the following two criteria: more than seven days difference in menstrual cycle duration between two consecutive cycles or, presence of a menstrual cycle duration over sixty days and progressive elevation of FSH levels.

### 2.5. Participants in Menopause Pilot Study

Menopause was confirmed according to the National Institute for Health and Care Excellence (NICE) guidelines [10]. The inclusion criteria for recruitment consisted of women’s age from 45 years to 55 years old, amenorrhea for at least 12 months without hormone replacement (HR) supplementation, and elevated levels of FSH > 30 IU/L.

For the POI, perimenopausal and menopausal cohorts, following recruitment and prior to PRP treatment participants completed the standard examination as described below. Following PRP treatment participants underwent follow-up monitoring as described below, and were invited to conceive naturally via regular unprotected sexual intercourse.

### 2.6. Standard Examinations Prior to Platelet-Rich Plasma Treatment

Regarding standard examinations on the participants’ reproductive dynamic, AFC was evaluated by transvaginal ultrasonography (TVUS) and FSH, luteinizing hormone (LH), AMH and E_2_ levels were considered markers indicating ovarian functionality. Regarding the POR cohort, FSH, LH and AMH levels analysis was performed on day 3 of the menstrual cycle. This was the menstrual cycle during which they underwent the ICSI-ET cycle prior to PRP treatment. When leading follicles were ≥18 mm this indicated that the patient could be then subjected to hCG triggering to enable the final maturation stage prior to oocyte retrieval. At that point E_2_ levels were evaluated and hCG was administrated on the evening of the same day. In this study, authors considered evaluation of E_2_ levels, at the day of hCG triggering, as an additional marker indicating the extent of a successful ovarian stimulation. Regarding the perimenopausal cohort, evaluation of FSH, LH, E_2_ and AMH levels, was performed on day 3 of the menstrual cycle. In the cases of amenorrhea for POI and menopausal cohorts, FSH, LH, E_2_ and AMH levels were evaluated on a random day. Furthermore, prior to PRP treatment all women were subjected to hysterosalpingography in order to evaluate patency of the fallopian tubes, the uterine cavity, and to exclude any other anatomical pathology or presence of a hydrosalpinx. In regard to the male partners all were subjected to semen analysis in order to exclude cases of male factor infertility.

FSH and LH levels analysis was performed by the chemiluminescent microparticle immunoassay on a Roche Immunoanalyser (Roche Cobas e 411, Basel, Switzerland). E_2_ levels analysis was performed employing the chemiluminescent microparticle immunoassay on a Roche Immunoanalyser (Roche Cobas e 411, Basel, Switzerland). AMH serum levels analysis was performed employing AMH PLUS chemiluminescent microparticle immunoassay (Roche Diagnostics GmbH, Mannheim, Germany) on a Roche Immunoanalyser (Roche Cobas e 411, Basel, Switzerland).

With respect to AFC, TVUS was performed on day 3 of the menstrual cycle. In cases of amenorrhea, AFC was recorded employing TVUS on a random day. TVUS was performed employing a GE Logiq P5 ultrasound system (GE Healthcare, Athens, Greece) armed with a vaginal three-dimensional probe (6.5–9 MHz). Assessment of each follicle was performed independently, accounting for both the longitudinal and transverse axis. Antral follicles were described as an ultrasound evaluation within the ovarian parenchyma of an anechoic image with dimensions ranging from 2 to 10 mm.

### 2.7. Platelet-Rich Plasma Preparation Method

According to the protocol employed, PRP administration took place during the early follicular phase of the cycle. For women presenting with menstrual cycles, such as POR and perimenopausal women that was day 3 of the menstrual cycle. For POI and menopausal women being amenorrheic, PRP administration was performed on a random day. With respect to preparation timing, PRP was always prepared earlier on the day of administration for all groups. Preparation of PRP was performed immediately following blood sample collection. Blood samples were collected from the median antebrachial vein. PRP was prepared according to the manufacturer’s instructions employing a RegenACR^®^-C Kit (Regen Laboratory, Le Mont-sur-Lausanne, Switzerland). Approximately 60 mL of the patient’s peripheral blood was required in order to yield the required volume of PRP. The initial concentration of platelets in peripheral blood was approximately 250,000 platelets/μL. The goal concentration of platelets in PRP was approximately 1,000,000 platelets/μL. According to our protocol, prepared PRP could be stored for one hour at a temperature of 4 °C, if required. However, regarding the vast majority of the participants, PRP intraovarian infusion was performed immediately following preparation.

### 2.8. Platelet-Rich Plasma Intraovarian Infusion Method

Regarding all four pilot studies, participants who did not receive HR were scheduled to undergo PRP treatment immediately following standard investigation. Participants who received HR treatment were invited to discontinue this treatment for at least six months prior to PRP treatment. Following the six months period, these participants were submitted to the standard investigation, as described above.

**Figure 1:**
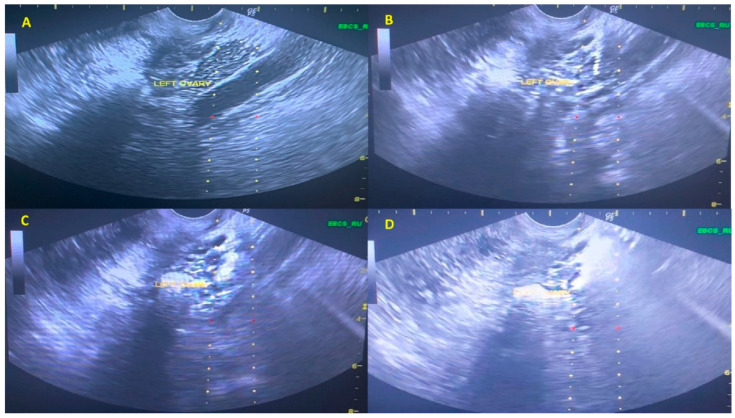
Sequence of images (**A**–**D**), illustrating the steps during PRP procedure on the left ovary. (**A**): Transvaginal Ultrasound Scan Image of the left ovary and the needle guide viewed prior to needle insertion. (**B**): Transvaginal Ultrasound Scan Image of the left ovary while the needle is fully inserted into cortex of the ovary. (**C**): Transvaginal Ultrasound Scan Image of the left ovary during PRP injection. (**D**): Transvaginal Ultrasound Scan Image of the left ovary aligned with the needle guide, following PRP injection.

Following PRP preparation, the technique of injection can mainly be described as an empirical approach, resembling the transvaginal paracentesis performed during the oocyte pick-up procedure, as previously described in detail by Pantos and colleagues [34]. Briefly, both ovaries were visualized via transvaginal ultrasound monitoring, and they were intramedullary injected on multiple sites using a 17-gauge single lumen needle, with the patient under inhaled minimal sedation. The technique included penetration across the central part of each ovary respectively, and thereafter gradual infusion of 4 mL of activated PRP, via a syringe attached to the transvaginal probe transducer. Following the infusion procedure, the pelvis was thoroughly examined via ultrasonography, in order to check total vascular integrity. Minor leakage was observed following retraction of the needle employed. Participants were recommended to remain in supine position for almost 15 min. The PRP infusion procedure is presented in Figure 1.

### 2.9. Follow-Up Monitoring

The duration of the follow-up period was three months. During this period, assessment of ovarian function employing evaluation of AFC, AMH, FSH, LH, and E_2_ levels was conducted for three constitutive menstrual cycles. Hormonal analysis, including FSH, LH, E_2_ and AMH levels, was performed on day 3 of the menstrual cycle. Regarding the POI and menopausal cohorts, in case of failure to restore menstrual cycle within the time-frame of three months following treatment and amenorrhea persisting, AFC and hormonal profile were evaluated monthly following PRP infusion.

Following PRP infusion, the treatment was regarded as successful if women following treatment failed to be re-classified as POR, POI, perimenopausal or menopausal, respectively.

Considering the POR cohort, the primary outcome measure was AFC, AMH levels, and oocyte yield in the ICSI-ET cycle post treatment. The secondary outcome measures were mature metaphase II (MII) oocyte yield in the ICSI-ET cycle post PRP, number of resulting embryos, and cycle cancellation rate.

Regarding the perimenopausal cohort, the primary outcome measures entailed restoration of menstrual cycle regularity, defined as less than seven days difference in menstrual cycle duration between two consecutive cycles and presence of a menstrual cycle duration less than sixty days, as well as FSH levels. Restoration of menstrual cycle regularity was recorded as a positive response to PRP for perimenopausal women. The reported secondary outcome measures were AFC, AMH, LH, and E_2_ levels.

Regarding the POI and menopausal groups, the primary outcome measures entailed restoration of menstrual cycle as well as FSH levels. Menstrual cycle restoration and FSH decrease were recorded as a positive response to PRP for POI and menopausal women. The reported secondary outcome measures were AFC, AMH levels, and E_2_ levels.

### 2.10. Statistics

All data analysis was performed using the R Programming Language for Statistical Purposes [38]. Due to the great volume of data presented herein and in order to ensure consistency with respect to data presentation, descriptive statistics are presented as mean (M) and standard deviation (SD) concerning all values, regardless of the distribution they followed. However, statistical analysis was performed according to the distribution of the values, employing parametric tests regarding normally distributed values, and non-parametric tests regarding values lacking normal distribution. Considering the limited number of participants included in each of the study’s group (*n* = 30), the Kolmogorov-Smirnov normality test was employed in order to assess whether the tested data originated from a normally distributed population.

To detect any difference the non-parametric Friedman test and Nemenyi post-hoc analysis was performed regarding the POR group, and the Generalized Linear Mixed Models regarding POI, menopausal and perimenopausal groups. Furthermore, the non-parametric Wilcoxon signed-rank test was employed to compare the clustered-paired outcomes prior and following PRP treatment. Regarding, normally distributed values the parametric paired Student’s *t*-test was employed.

Confidence intervals of 95% were calculated for each variable, and a *p* value < 0.05 was considered to be statistically significant.

### 2.11. Study Approval

The Ethics Board of Centre of Human Reproduction approved the pilot studies’ protocols and the consent forms (Registration Number: 17/10-1-20170) in accordance with the Helsinki declaration [39]. All women eligible to participate in the pilot studies were subjected to extensive consultation regarding the studies’ protocols. Participation was voluntarily, providing oral and written informed consent. In particular, following initial screening and assessment of eligibility for recruitment, all patients were subjected to thorough consultation. During this process patients were provided with two copies of the respective informed consent form and were encouraged to present any questions pertaining to the study. Finally, they were asked to carefully study the informed consent form at their own time prior to returning. The informed consent forms clearly stated that intraovarian PRP is an experimental therapeutic approach and it is not considered a clinical routine practice. Further to that, the informed consent form provided information pertaining to possible risks potentially associated with the procedure that the patients were asked to acknowledge. Patients that decided to participate returned a signed copy of the informed consent form to the clinic.

## 3. Results

### 3.1. Platelet-Rich Plasma Preparation and Infusion

PRP was successfully prepared following blood collection regarding all of 120 participants recruited. All patients reached the threshold of 1,000,000 platelets/μL following PRP preparation. Autologous PRP intraovarian infusion was successfully implemented regarding all of the study’s participants. None of the study’s participants presented with any adverse effect, indicating that intraovarian PRP infusion may be viewed a safe technique according to results sourced herein.

### 3.2. Results Following Platelet-Rich Plasma Intraovarian Infusion in Women Presenting with Poor Ovarian Response

**Figure 2:**
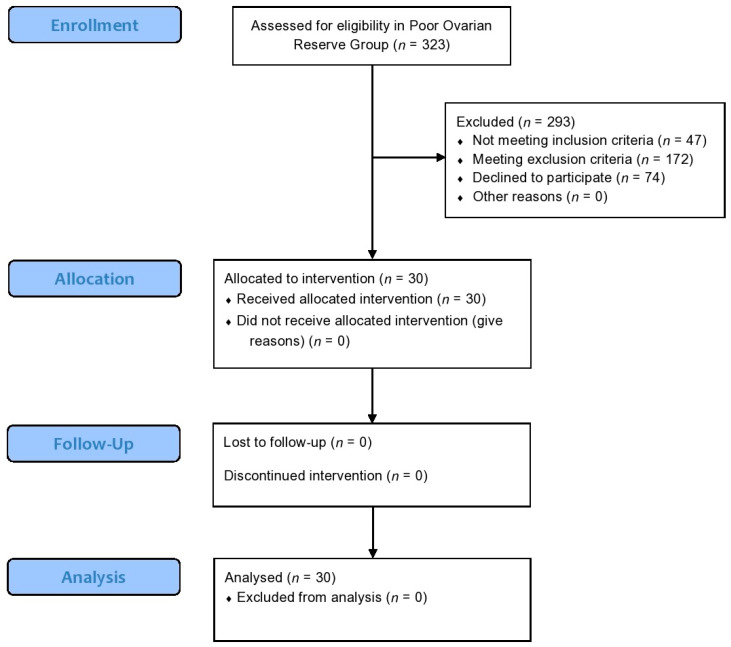
A modified version of CONSORT flow-chart presenting enrollment, allocation, follow-up and analysis of participants regarding the Poor Ovarian Reserve pilot study.

From February 2017 to January 2018, a total of 323 women were screened for the study and assessed for eligibility. A detailed outline on participants’ enrollment, allocation, follow-up and analysis regarding the POR pilot study is provided in Figure 2.

Results regarding PRP efficiency on the POR pilot study are presented in detail in Table 2. FSH and LH levels were reduced in the first menstrual cycle following PRP treatment and remained stable in the second and the third menstrual cycle. AMH levels as well as AFC increased in the first menstrual cycle following PRP, further increased in the second menstrual cycle and remained stable in the third. Further to that, as described in detail below, ICSI-ET cycle performance post PRP treatment was considerably improved with regard to mature oocyte yield, and cleavage stage formation rate, leading to improved clinical and live birth rates and a reduced cancellation rate.

Regarding the ICSI-ET cycle prior to PRP treatment, out of the 30 participants subjected to COS, oocyte retrieval was successfully performed for 25 (83.3%). In total, 36 oocytes were retrieved from 30 participants who underwent COS, resulting in 30 mature MII oocytes. In total, 12 cleavage embryos were transferred. Clinical pregnancy was not achieved for any of the participants.

In the ICSI cycle post PRP treatment, oocyte retrieval was successfully performed for 29 out of the 30 participants (96.6%). In total, 101 oocytes were retrieved from 30 participants who underwent COS. Six participants were subjected to single ET and 15 patients were subjected to double ET. Clinical pregnancy was achieved for 14 participants (46.6%). All pregnancies were singletons. Two spontaneous miscarriages were recorded for two participants, at the 9th and 11th week of gestation, respectively. Twelve participants achieved a live birth following complication-free pregnancies to term. Normal first and second trimester prenatal screening tests, along with reactive non-stress tests (NST) were observed.

In summary, comparing the post PRP ICSI-ET cycle with the one prior to PRP, a significant increase was observed in favor of the post PRP cycle. This increase pertained to the number of oocytes retrieved, the number of MII oocytes obtained, the number of two pronuclei embryos obtained, and the number of embryos reaching cleavage stage. Furthermore, cancellation rate was significantly decreased. Interestingly, a significant reduction was observed in regard to the duration of stimulation period. No statistically significant difference was observed regarding the total dose of gonadotropin administrated as well as regarding the number of good quality embryos obtained (Table 2).

### 3.3. Results Following Platelet-Rich Plasma Intraovarian Infusion in Women Presenting with Premature Ovarian Insufficiency

**Figure 3:**
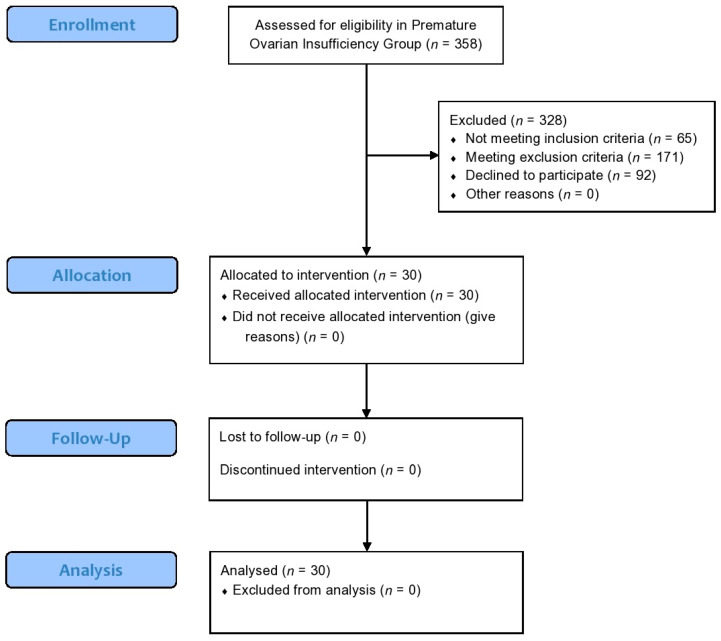
A modified version of CONSORT flow-chart presenting enrollment, allocation, follow-up and analysis of participants regarding the Premature Ovarian Insufficiency pilot study.

From February 2017 to May 2018, a total of 358 women were screened for the study and assessed for eligibility. A detailed outline on participants’ enrollment, allocation, follow-up and analysis regarding the POI pilot study is provided in Figure 3.

Results regarding PRP efficiency on the POI pilot study are presented in detail in Table 3. Eighteen women (60%) positively responded to PRP treatment, constituting what is herein described as the PRP success subgroup for this pilot study. These women presented with menstrual cycle restoration as well as reduced FSH levels. In 12 patients (40%), treatment was considered to be unsuccessful as amenorrhea persisted post PRP treatment throughout the follow-up period. These participants constituted what is herein described as the PRP failure subgroup.

Regarding the PRP success subgroup, AMH, E_2_ and LH along with AFC presented statistically significantly improved from the first moth post PRP treatment, while FSH similarly presented improved post PRP treatment with decreased levels noted from the second month of the follow-up. The failure subgroup presented with amenorrhea and no improvement on AFC. Nonetheless, a degree of improvement for the failure subgroup was noted regarding levels of FSH, LH, AMH and E_2_. Despite this improvement its extent was not as noteworthy as the one noted for the success subgroup, as presented in Table 3.

Comparison of the two subgroups’ characteristics prior to PRP treatment indicated no statistically significant difference in regard to participants’ age, partners’ age, and duration of amenorrhea period. Interestingly, baseline FSH levels prior to PRP treatment were significantly lower in the PRP success subgroup (Table 3).

Following PRP treatment, three participants included in the PRP success subgroup achieved natural conceptions. All pregnancies were singletons. All of them achieved a live birth following complication-free pregnancies to term. Normal first and second trimester prenatal screening tests, along with reactive NST tests were reported for all patients.

### 3.4. Results Following Platelet-Rich Plasma Intraovarian Infusion Regarding Women in Perimenopause

**Figure 4:**
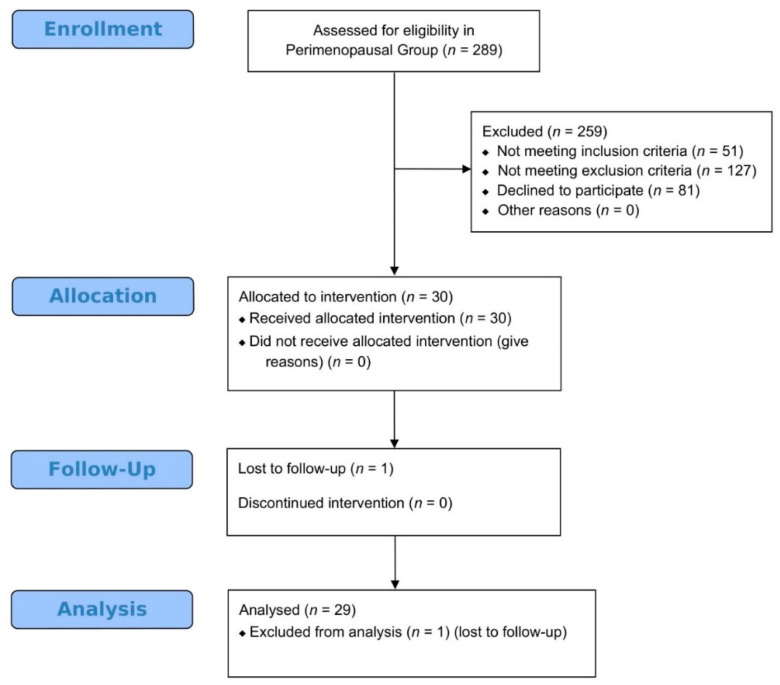
A modified version of CONSORT flow-chart presenting enrollment, allocation, follow-up and analysis of participants regarding the Perimenopausal pilot study.

From February 2017 to January 2018, a total of 298 women were screened for the study and assessed for eligibility. A detailed outline on participants’ enrollment, allocation, follow-up, and analysis regarding the perimenopausal pilot study is provided in Figure 4.

Results regarding PRP efficiency on the perimenopausal pilot study are presented in detail in Table 4. Twenty-four women (80%) positively responded to PRP treatment, constituting what is herein described as the PRP success subgroup for this pilot study. For these women menstrual cycle regulation as well as FSH level reduction was observed. Menstrual cycle regulation was defined as less than seven days difference in menstrual cycle duration between two consecutive cycles and presence of a menstrual cycle duration less than sixty days. In five women (16.6%), treatment was considered to be unsuccessful as menstrual cycle irregularities still persisted post PRP treatment throughout the follow-up period. These participants constituted what is herein described as the PRP failure subgroup.

Regarding the PRP success subgroup, AFC along with AMH and E_2_ presented statistically significantly improved from the first month of the follow-up post PRP treatment, while FSH and LH similarly presented improved post PRP treatment with decreased levels noted from the second follow-up month. The failure subgroup presented with menstrual cycle irregularities and no improvement in regard to any outcome measures throughout the follow-up post PRP, as presented in Table 4.

Comparison of the two subgroups’ characteristics prior to PRP treatment indicated no statistically significantly difference regarding participants’ age, duration of irregular period, menstrual cycle duration and menstruation duration. Mean partners’ age was significantly higher in the PRP failure group compared to the PRP success group (Table 4).

Following PRP treatment, four participants, included in the PRP success subgroup, achieved natural conceptions. All pregnancies were singletons. One spontaneous miscarriage was recorded at week 13 of gestation. Three participants achieved a live birth following complication-free pregnancies to term. Normal first and second trimester prenatal screening tests, along with reactive NST tests were recorded for all participants.

### 3.5. Results Following Platelet-Rich Plasma Intraovarian Infusion Regarding Women in Menopause

**Figure 5:**
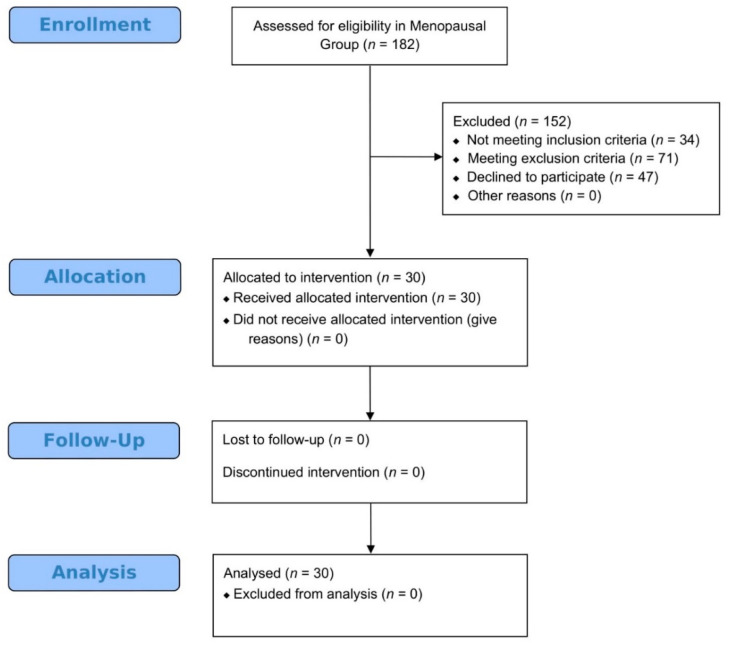
A modified version of CONSORT flow-chart presenting enrollment, allocation, follow-up and analysis of participants regarding the Menopausal pilot study.

From February 2017 to July 2018, a total of 182 women were screened for the study and assessed for eligibility. A detailed outline on participants’ enrollment, allocation, follow-up, and analysis regarding the menopausal pilot study is provided in Figure 5.

Results regarding PRP efficiency on the menopausal pilot study are presented in detail in Table 5. Thirteen women (43.3%) positively responded to PRP treatment, constituting what is herein described as the PRP success subgroup for this pilot study. These women presented with menstrual cycle restoration as well as reduced FSH levels. In 17 women (56.7%), treatment was considered to be unsuccessful as amenorrhea persisted post PRP treatment throughout the follow-up period. These participants constituted what is herein described as the PRP failure subgroup.

Regarding the PRP success subgroup, AFC along with AMH, FSH, LH and E_2_ presented statistically significantly improved from the first month of the follow-up post PRP treatment. The failure subgroup presented with amenorrhea and no improvement on AFC and E_2_. Nonetheless, a degree of improvement was noted for the PRP failure subgroup regarding AMH, FSH and LH. Despite this improvement its extent was not as noteworthy as the one noted for the success subgroup as clearly seen in Table 5.

Comparison of the two subgroups’ characteristics prior to PRP treatment indicated no statistically significantly difference regarding partners’ age and duration of the amenorrhea period. Women included in the PRP failure subgroup were presenting with higher mean age compared to women included in the success group (Table 5).

Following PRP treatment, one participant, included in the PRP success subgroup, achieved a natural conception, leading to a live birth, following a complication-free pregnancy in term. Normal first and second trimester prenatal screening tests, along with NST were recorded.

## 4. Discussion

Irrespectively of the etiology inducing collapse of ovarian function, the lack of stimulable primordial follicles constitutes the main origin of ovarian failure [2]. Furthermore, functionality of the ovarian niche is jeopardized and thus is unable to support effectively proliferation and differentiation of granulosa cells [40,41].

In addition, studies demonstrate that aging, POR and POI are conditions related to a reduced ovarian blood flow [42,43,44,45,46]. The vascular endothelial growth factor A (VEGF-A) seems to be the master regulator of the angiogenetic processes during folliculogenesis. VEGF-A production is mediated by the effect of several growth factors and hormones on granulosa cells. Among these growth factors and hormones are the insulin-like growth factor 1 (IGF-1), the growth hormone (GH), the gonadotropins, several steroid hormones, the fibroblast growth factor 2 (FGF-2), and thrombospondin (THSP-1) [47,48,49,50,51,52]. This molecular network promoting angiogenesis is reported to be significantly disrupted in patients presenting with ovarian insufficiency [53]. This deficiency in vascularization could be another factor contributing to impairment of ovarian functionality [42,43,44,45,46].

Considering the aforementioned, the pathophysiological base of ovarian insufficiency involves degenerative phenomena leading to collapse of the ovarian niche and disruption of the molecular network controlling ovarian vascularization. As a result, oxygen, nutrients, and hormonal supply is interrupted and follicular growth is compromised. However, studies indicate that this condition may be reversible and follicular growth could be stimulated when the jeopardized ovarian microenvironment is restored [31,32,33,34,35,53,54,55].

Numerous observations with respect to the angiogenetic structure of the ovaries, along with the vascular activation that is promoted by several platelet-derived factors [56], encouraged our team of experts to test the hypothesis that autologous PRP intraovarian infusion treatment may be able to induce reactivation of dysfunctional ovarian tissue [30]. In fact, it its highlighted that PRP may play an important role in ovarian niche restoration, mainly through the promotion of the physiological processes of angiogenesis, proliferation and growth, apoptosis, inflammation control, as well as cell migration [20,21,54,57]. Considering the regenerative potential of PRP treatment, these pilot studies were conducted aiming to provide data investigating the potential of PRP treatment application toward rejuvenating ovarian function in women presenting with different types of ovarian insufficiency.

Results presented herein indicate that PRP intraovarian infusion could effectively promote folliculogenesis and restore ovarian functionality and hormonal profile. This was the case for all four groups investigated as evidenced from the progressively increased AFC, AMH and E_2_ levels, while similarly, FSH and LH levels progressively decreased. Hence, PRP treatment led to enhancement of Hypothalamus Pituitary Adrenal (HPA) axis’ sensitivity sometimes as early as from the first month following PRP treatment. In particular, this improvement was noted for all POR participants, for the vast majority of perimenopausal participants and for a considerable percentage of the POI and menopausal participants. Furthermore, it is noteworthy that a significant percentage of perimenopausal participants experienced regularity in menstrual cycles following PRP. Similarly, for a great percentage of our POI and menopausal women, menstrual cycle restoration was achieved.

Regarding the POR group, PRP treatment resulted in an increased number of oocytes retrieved and MII oocytes obtained following the ICSI cycle post PRP. As anticipated, the number of embryos obtained was also increased. However, data presented herein do not support that PRP treatment exerted any positive influence on the quality of embryos obtained. It is of paramount importance to note that the cancellation rate was significantly reduced following PRP. Interestingly, following PRP, COS duration was significantly reduced and COS performance was significantly improved even though the total dose of gonadotropin administration was the same. Twelve of our patients achieved live births following a single ICSI cycle post PRP treatment. All pregnancies presented to term, while no obstetrical, or perinatal complications were reported for any of the patients.

The scarcity of stimulable follicles in POR patients, results in low oocyte yield and high cancellation rates [2]. Despite advances, epidemiological studies indicate that POR women undergoing ART present with an overall 18.7% clinical pregnancy rate, and 23.6% cycle cancellation rate [58]. IVF success rate in POR patients is significantly compromised in comparison to the overall rates ranging from 30–35%. Interestingly, literature indicates that only 33% of patients with POR who fail in the first IVF/ICSI attempt pursue a second cycle [59]. PRP’s soluble factors which promote neo-angiogenesis, enable an ovarian environment in supporting the growth of small secondary pre-antral follicles. These follicles can respond to exogenous gonadotropins, resulting in larger, ovulatory, antral follicles. At this point it should be highlighted that the patients presented herein were young POR patients (mean age 38.40 years old) and thus the efficiency of PRP treatment in advanced age (>40 years old) POR patients cannot be accounted for in the present study. Interestingly, recent data demonstrated that PRP intraovarian infusion may improve ovarian reserve in POR patients of advanced age [60]. Melo and colleagues showed a significant improvement in AMH levels and AFC during the first three months of the follow-up period, while participants presented with a higher clinical pregnancy rate compared to the control group [60]. In our study, we report an overall clinical pregnancy rate of 46.6% following PRP treatment. Our participants were on average three years younger, and subjected to ICSI treatment on the third month following a single PRP treatment in contrast to the study of Melo et al., 2020 in which patients were subjected to ART treatment within a 12-month time-frame [60]. Considering results presented in the study of Melo et al., 2020 as well as in ours, it seems that PRP treatment may be effective in POR patients of both young and advanced age. However maternal age may strongly impact on final pregnancy outcomes [60].

Considering the POI group, PRP treatment resulted in menstrual cycle restoration for 60% of the participants. Three of our POI patients achieved natural conceptions and respective live births, following complication-free pregnancies at term.

It should be noted that a significant proportion (40%) of our POI patients remained amenorrheic with no AFC detected, albeit AMH levels increased, and FSH and LH levels decreased. It appears that PRP treatment improved the parameters reflecting on ovarian reserve as well as these indicating sensitivity of the HPA axis, nevertheless, folliculogenesis was not induced. To investigate this paradox, we compared patients’ performance between the PRP success and failure groups, and we observed significantly higher AMH levels and lower FSH and LH levels in the success group. It is well established that in POI patients, primordial follicle loss is pathologically accelerated. However, recent studies demonstrate that a significant proportion of POI patients’ primordial follicles remain dormant [61,62]. One possible explanation of this paradox is that PRP treatment effectively restored the ovarian niche environment. This may entail promoting primordial follicle development up to the antral stage in our POI patients who achieved menstrual cycle restoration, leading to AMH and E_2_ increase and concurrently to FSH and LH decrease. Regarding our POI patients who were still presenting with amenorrhea, PRP treatment also promoted primordial follicle development. However, their development failed to reach the antral stage, leading to a significant lower increase in AMH levels and to a lesser decrease in FSH and LH levels. It is well documented that AMH levels are mainly associated with the number of medium-sized antral follicles (5–8 mm) of the ovaries [63]. However, recent data demonstrate that AMH levels are also related to the number of small follicles (<5 mm). It may be extrapolated that the granulosa cells present in these small pre-antral follicles-failing to reach the antral stage-produced AMH, leading to the respective increase of AMH levels in PRP failure POI group [63].

Attempts to address POI were hitherto focused on improving ovarian function employing HR [64,65]. Even if POI patients are treated effectively with HR, IVF/ICSI success is significantly low [66]. Similarly, considering surrogacy or adoption social, ethical, economic, and legal issues may be raised [12,67]. The vast majority of POI patients opt for oocyte donation [15]. However, these options are not always welcomed by patients [68]. Undoubtedly ovarian insufficiency and its treatment or rather the lack of a universally accepted line of approach, remain a conundrum for practitioners. PRP intraovarian infusion appears to enable menstrual cycle recovery and ovarian function restoration, promoting the physiological process of folliculogenesis, as well as restoring the hormonal profile of some POI patients.

The efficiency of intraovarian autologous PRP infusion was also investigated in perimenopausal and menopausal women. In these categories of women, ovarian insufficiency is attributed to the physiological process of aging. Regarding the perimenopausal group, PRP treatment resulted in restoration of menstrual cycle regularity for 80% of the participants. Interestingly, four perimenopausal women achieved natural conceptions. One spontaneous miscarriage was reported. Three patients achieved a live birth following complication-free pregnancies to term.

During perimenopause the number of stimulable follicles restricted and granulosa cells are unable to effectively respond to gonadotropins [69]. Perimenopause is commonly treated with oral contraceptives or hormonal contraceptives in patch or ring forms aiming to alleviate symptoms [70]. However, if treatment is discontinued symptoms rebound. When a high risk of cardiovascular disease, is implicated in perimenopause this serves as a contraindication for HR treatment which should be avoided as the risk of myocardial infarction and stroke rises with age, and exposure to hormonal contraception increases the relative risk of an adverse cardiovascular event [70]. Our data support that PRP treatment increases the number of stimulable follicles leading to menstrual cycle regularity and to hormonal profile restoration in perimenopause. This may in turn relief menopause-related symptoms while enabling fertility potential, rendering oocyte donation as a last resort option.

In regard to the menopausal group, menstrual cycle restoration was achieved for 43.3% of the studied population. One of our menopausal patients achieved a natural conception leading to live birth.

It should be noted that 56.7% of our menopausal women failed to respond to PRP treatment and remained amenorrheic. Menopausal women presented with a rather similar performance to POI patients regarding both the success and failure groups for either category, despite apparent differences. Similarities pertained to FSH, LH, AMH and AFC, with success groups showcasing a better response. This indicates that compromise of the ovarian niche, as anticipated, was heightened for these two groups in comparison to perimenopausal and POR patients. Following on that the dynamic for rejuvenation of ovarian function was lesser in comparison to POR and perimenopausal groups. The biologically paradox phenomenon of hormonal profile improvement-evident in both menopausal and POI groups-is of a great interest and merits molecular investigation. The traditional principle on the loss of ovarian function due to aging, supports that at birth the follicular number is fixed and is subject to decrease thence after and following menarche, until it is depleted in menopause. However, data demonstrate the existence of ovarian stem cells [41]. During the female reproductive lifespan, these proliferative ovarian stem cells differentiate into ovarian reproductive cells via asymmetrical division, promoting ovarian regeneration and folliculogenesis [41]. Menopause occurs when the proliferation and the differentiation of the ovarian stem cells is compromised, mainly due to the jeopardized vascularization of the ovary [41]. Following menopause there is still several inactive primordial follicles that may be stimulated following ovarian niche restoration as the PRP success group indicates [71,72]. Regarding menopausal patients still presenting with amenorrhea, PRP treatment also promoted primordial follicle development; however this process stopped prior to reaching the antral stage. It is this that may have led to a significantly lesser increase in AMH levels and to a diminished decrease in FSH and LH levels. Till date, women in menopause embark on HR aiming for relief from vasomotor symptoms and other menopause-related conditions [73,74]. The authors refrain from attempting a comparison of PRP application to other widely accepted and applied approaches of routine clinical practice status. Nonetheless, PRP treatment may be present as an alternative toward addressing the menopause-related symptoms.

Considering data provided herein, intraovarian PRP infusion appears to be effective for some of the recruited patients. As specific criteria were applied in order to achieve homogeneity in studied populations, this study fails to provide driving characteristics distinguishing between responsiveness or unresponsiveness to PRP treatment. However, statistical analysis comparing the ‘’success’’ and ‘’failure’’ subgroups revealed that parameters, including patients’ age and basal FSH levels—for the menopausal and POI groups respectively—could have prognostic value in regard to PRP treatment’s outcome. It maybe so that younger and of lower FSH levels participants may be more responsive to treatment. For these reasons, it is imperative to investigate this treatment’s efficiency in the context of personalized medicine.

Considering POI and POR patients, it is an absolute requirement to specify the exact pathophysiological etiology causing ovarian insufficiency. Furthermore, considering perimenopausal and menopausal women, it is of high significance to address the patient’s actual biological age and to assess reproductive capacity in order to evaluate PRP’s therapeutic potential [75]. Managing different pathologies may require different optimal concentrations of platelets. This could be another factor leading to heterogeneity of results and outcomes of the current study [76]. Several other factors, potentially affecting treatment’s outcome should be elucidated in future studies focusing on optimal protocol design. There are questions pertaining to the number of PRP infusions that constitute one treatment cycle, time intervals between infusions-especially if administrations are consecutive as in the case of Melo et al., 2020 [60], with optimal time-frame between PRP infusions and IVF/ICSI treatment, volume administered, as well as platelets’ threshold. Future studies should follow a ‘’from bench to bedside’’ approach, to provide sufficient data investigating molecular and physiological mechanisms entailing the therapeutic value of PRP infusion in different types of ovarian insufficiency.

Despite PRP application being described as a straightforward procedure, nonetheless it entails predicted challenges. Intraovarian injection requires patient individualized standardization by the practitioner. Regarding certain categories of women such as menopausal women and POI patients, the practice of PRP infusion in atrophic ovaries of jeopardized volume could be a complex and demanding procedure [35]. Patients suffering from certain inherited platelet disorders [77], even thrombocytopenia, or those who are subjected to anti-inflammatory medication or even anticoagulants, along with smokers, bone or hematopoietic cancer patients, should be discouraged from PRP treatment, as its biological action could be hindered [33]. Considering intraovarian autologous PRP infusion as a future therapeutic option for ovarian insufficiency, it should be highlighted that the method presents with both strengths and limitations which should equally be considered prior to horizontal application in clinical practice. Till date, no study reports on short-term or long-term side effects. PRP represents an autologous product entailing a mild immune response. Thus, the possibility of potential side effects that are related to administration of a heterologous sample-such as graft rejection-is minimized. The concern on the probability of transmitting a contagious disease is limited [78]. Despite the fact that no association was demonstrated between PRP components and any tumor promotion [79], and that the intense cell proliferation events entailed may induce malignancy was voiced [55]. Although there is no evidence demonstrating any side effects regarding PRP application in the reproductive system, we may be far from reassuring patients on long-term safety [31,32,33,34,35,54,55].

Despite the encouraging results presented herein, the current study presents with serious limitations and has a high risk of bias. The absence of control groups, mainly attributed to the observational nature of this pilot study, is the main limitation, coupled with the small sample size. The relatively limited follow-up period could serve as a further limitation factor.

## 5. Conclusions

Data presented herein indicate that autologous intraovarian PRP infusion may restore ovarian function, enabling reactivation of the folliculogenesis process, recovery of menstrual cycle, and the enhancement of the hormonal profile. This may in turn enable achievement of pregnancy—even via natural conception—for certain women that are still exploring options on employing their own gametes. The questions “for whom”, “when”, “how often”, along with concurring on preparation method and administration technique of PRP still remain to be answered. Future studies are required in order to provide concrete evidence in search of the “holy grail” in managing ovarian insufficiency. It is imperative to conduct well-designed RCTs, in an effort to identify the profile of women who may benefit from the clinical application of PRP. Our team has already initiated conduction of four RCTs, regarding autologous infusion of PRP in poor responders (NCT03937661), in perimenopausal women (NCT03951194), in POI women (NCT04031456), as well as in menopausal women (NCT03916978). Till robust data are sourced and presented, intraovarian PRP infusion should be consider strictly as an experimental method and should not be viewed as a valid alternative option addressing ovarian insufficiency. It is of significance to exhaustively investigate the safety and efficacy of newly introduced therapies prior to offering these in clinical practice [36]. Further data may provide the final verdict.

## Figures and Tables

**Table 1:** Details regarding study design for the four pilot studies.

Pilot Study	Screening for Eligibility	Standard Examination Prior to PRP Treatment	PRP Intraovarian Infusion	Follow-Up Monitoring
POR (*n* = 30)	Bologna Criteria	- Assessment of AFC, serum FSH, LH, AMH and E_2_ - HSG and semen analysis	Performed at least 2 months following the last ICSI-ET cycle	- AFC, serum FSH, LH, AMH and E_2_ assessment for three consecutive months following PRP treatment - In the third month following PRP participants were subjected to an ICSI-ET cycle
POI (*n* = 30)	- Age <40 years - Amenorrhea for at least four months, and elevated FSH >25 IU/L	-Assessment of AFC, serum FSH, LH, AMH and E_2_ -HSG and semen analysis	Performed immediately following standard investigation or at least six months following HR discontinuation	- AFC, serum FSH, LH, AMH and E_2_ assessment for three consecutive months following PRP treatment. - Participants were invited to conceive naturally
Perimenopause (*n* = 30)	-Age ≥40 years -Menstrual cycle irregularities			
Menopause (*n* = 30)	- Age 45–55 years - Amenorrhea for at least 12 months without HR supplementation, and FSH >30 IU/L			

POR: Poor Ovarian Response; POI: Premature Ovarian Insufficiency; FSH: Follicle Stimulating Hormone; LH: Luteinizing Hormone; AMH; Anti-Müllerian Hormone; E_2_: Estradiol; AFC: Antral Follicle Count; HSG: Hysterosalpigography; HR: Hormone Replacement; PRP: Platelet-Rich Plasma; ICSI: Intracytoplasmic Sperm Injection; ET: Embryo Transfer.

**Table 2:** Descriptive statistics indicating participants’ general characteristics and respective performance prior to and following PRP treatment in the Poor Ovarian Response pilot study.

**Parameters**	**Poor Ovarian Response Group (*n* = 30)**			
	**General Participants’ Characteristics**			
Patient age (years)	38.40 ± 2.01			
Partner age (years)	40.9 ± 2.52			
Years of infertility	5.83 ± 1.02			
Body Mass Index (kg/m^2^)	23.12 ± 2.52			
**Ovarian Reserve Markers**	**Prior ICSI Cycle**	**1st Menstrual Cycle**	**2nd Menstrual Cycle**	**Post ICSI Cycle**
FSH (IU/mL)	10.71 ± 1.62	9.05 ± 1.76 a(**)	8.87 ± 1.68 a(**)	8.95 ± 1.40 a(**)
LH (IU/mL)	9.02 ± 0.80	7.10 ± 1.03 a(**)	6.50 ± 0.98 a(**)	6.08 ± 0.92 a(**)
AMH (ng/mL)	0.66 ± 0.20	0.85 ± 0.26 a(*)	1.17 ± 0.28 a(**),b(**)	1.14 ± 0.26 a(**),b(**)
AFC	2.63 ± 0.93	3.80 ± 1.06 a(*)	5.33 ± 1.32 a(**),b(**)	5.20 ± 1.35 a(**),b(**)
**ICSI Cycle’s Performance**	**Prior ICSI Cycle**	**1st Menstrual Cycle**	**2nd Menstrual Cycle**	**Post ICSI Cycle**
Dur. of stimulation (Days)	10.57 ± 0.90	NA	NA	9.40 ± 1.10 a(**)
Gonadotropin Dose (IU)	4234.30 ± 261.58	NA	NA	4316.70 ± 217.93
E_2_ (pg/mL) (hCG Trigger)	710.33 ± 226.36	NA	NA	1522.90 ± 472.02 a(**)
Retrieved Oocytes	1.20 ± 0.76	NA	NA	3.37 ± 1.54 a(**)
MII Oocytes Obtained	1.00 ± 0.79	NA	NA	2.97 ± 1.38 a(**)
2PN Embryos Obtained	0.73 ± 0.52	NA	NA	2.43 ± 1.38 a(**)
Cleavage Stage Embryos	0.60 ± 0.56	NA	NA	1.93 ± 1.26 (**)
Total Number of Cleavage Stage Embryos	18	NA	NA	58
Good quality (Grade 1 & 2)	8/18 (44.4%)	NA	NA	28/58 (48.2%)
Cancellation Rate	19/30 (63.3%)	NA	NA	9/30 (30%) a(*)

a: Indicate ICSI Cycle Prior to PRP. b: Indicate 1st Month Following PRP; *: Indicate level of significance corresponding to *p* value < 0.05; **: Indicate level of significance corresponding to *p* value < 0.001; NA: Not applicable.

**Table 3:** Statistical analysis and outcome assessment prior and following PRP intraovarian infusion regarding the Premature Ovarian Insufficiency pilot study.

**General Participants’ Characteristics**	**PRP Success (*n* = 18)**					**PRP Failure (*n* = 12)**					***p* Value**	
Participant age (years)	35.11 ± 1.57					35.92 ± 1.93					NS	
Partner age (years)	37.94 ± 1.21					37.88 ± 1.70					NS	
Duration of amenorrhea (months)	10.06 ± 2.62					10.17 ± 4.76					NS	
Time to menstrual cycle recovery (days)	42.06 ± 6.43					NA					NA	
**Outcome Measures Following PRP**	**Prior to Treatment**			**Follow-Up Month 1**			**Follow-Up Month 2**			**Follow-Up Month 3**		
	**PRP Success**	**PRP Failure**	***p* Value**	**PRP Success**	**PRP Failure**	***p* Value**	**PRP Success**	**PRP Failure**	***p* Value**	**PRP Success**	**PRP Failure**	***p* Value**
Menstrual cycle duration (days)	NA	NA	NA	34.22 ± 4.10	NA	NA	34.06 ± 4.33	NA	NA	33.44 ± 3.57	NA	NA
Menstruation duration (days)	NA	NA	NA	4.22 ± 1.31	NA	NA	4.56 ± 1.20	NA	NA	4.89 ± 1.18	NA	NA
FSH (IU/mL)	40.61 ± 6.05	63.65 ± 6.41	<0.001	35.87 ± 4.77	54.07 ± 8.98	<0.001	27.18 ± 5.82	52.47 ± 8.09	<0.001	20.67 ± 3.58	59.40 ± 9.47	<0.001
LH (IU/mL)	25.14 ± 3.10	24.33 ± 3.04	NS	21.91 ± 2.00	22.23 ± 2.38	NS	20.11 ± 1.45	17.51 ± 2.53	NS	19.31 ± 1.93	23.50 ± 4.37	0.001
AMH (ng/mL)	0.18 ± 0.04	0.15 ± 0.04	NS	0.53 ± 0.10	0.21 ± 0.06	<0.001	0.65 ± 0.08	0.27 ± 0.09	<0.001	0.75 ± 0.06	0.30 ± 0.05	<0.001
E_2_ (pg/mL)	17.13 ± 2.22	17.38 ± 2.61	NS	26.75 ± 4.56	23.43 ± 2.86	NS	38.92 ± 9.46	25.14 ± 1.99	<0.001	48.08 ± 6.28	20.86 ± 7.11	<0.001
AFC	0	0	NS	1.56 ± 0.51	0	<0.001	2.06 ± 0.73	0	<0.001	2.33 ± 0.49	0	<0.001
**Spontaneous Pregnancies Recorded Following PRP**	**PRP Success**					**PRP Failure**					***p* Value**	
Number of Pregnancies achieved	3					0					NS	
Number of Live Births	3					0					NS	

NA: Not applicable; NS: No statistically significant difference.

**Table 4:** Statistical analysis and outcome assessment prior and following PRP intraovarian infusion regarding the Perimenopausal pilot study.

**General Participants’ Characteristics**	**PRP Success (*n* = 24)**					**PRP Failure (*n* = 5)**					***p* Value**	
Participant age (years)	43.25 ± 1.42					44 ± 2.55					NS	
Partner age (years)	44.92 ± 3.23					49 ± 2.00					<0.001	
Menstrual cycle irregularities (months)	16 ± 2.43					17 ± 2.35					NS	
**Outcome Measures Following PRP**	**Prior to Treatment**			**Follow-Up Month 1**			**Follow-Up Month 2**			**Follow-Up Month 3**		
	**PRP Success**	**PRP Failure**	***p* Value**	**PRP Success**	**PRP Failure**	***p* Value**	**PRP Success**	**PRP Failure**	***p* Value**	**PRP Success**	**PRP Failure**	***p* Value**
Differences in menstrual cycle duration between two consecutive cycle (days)	10.08 ± 2.10	12.00 ± 1.58	NS	NA	NA	NA	0.79 ± 0.66	12.40 ± 3.91	<0.001	1.29 ± 1.27	10.20 ± 2.68	<0.001
Menstrual cycle duration (days)	41.13 ± 1.85	47 ± 10.42	NS	37.08 ± 5.32	40.80 ± 8.61	NS	36.88 ± 5.62	36.80 ± 9.36	NS	36.75 ± 4.93	38.60 ± 4.88	NS
Menstruation duration (days)	3.17 ± 1.17	3.60 ± 1.52	NS	3.92 ± 1.72	4.40 ± 1.52	NS	5.33 ± 1.69	4.60 ± 1.95	NS	4.92 ± 1.38	5.00 ± 1.41	NS
FSH (IU/mL)	18.51 ± 2.62	18.32 ± 1.78	NS	17.03 ± 3.57	17.82 ± 1.41	NS	15.10 ± 3.65	18.24 ± 1.89	NS	15.28 ± 4.03	18.14 ± 1.82	NS
LH (IU/mL)	16.28 ± 2.64	16.62 ± 1.97	NS	14.72 ± 3.43	15.90 ± 1.84	NS	13.20 ± 3.53	16.22 ± 1.80	NS	13.18 ± 3.52	16.30 ± 1.36	NS
AMH (ng/mL)	0.96 ± 0.28	0.86 ± 0.36	NS	1.42 ± 0.16	0.80 ± 0.37	<0.001	1.41 ± 0.17	0.82 ± 0.38	<0.001	1.41 ± 0.23	0.66 ± 0.36	<0.001
E_2_ (pg/mL)	29.67 ± 3.82	27.80 ± 6.87	NS	39.50 ± 2.06	28.70 ± 5.97	<0.001	39.29 ± 5.82	31.34 ± 7.14	0.026	40.83 ± 4.30	30.24 ± 9.86	<0.001
AFC	1.54 ± 0.51	1.00 ± 0.71	NS	2.79 ± 0.78	1.20 ± 0.45	<0.001	3.38 ± 0.92	1.40 ± 0.89	<0.001	4.25 ± 0.68	1.20 ± 1.10	<0.001
**Spontaneous Pregnancies Recorded Following PRP**	**PRP Success**					**PRP Failure**					***p* Value**	
Number of Pregnancies achieved	4					0					NS	
Number of Live Births	3					0					NS	

NA: Not applicable; NS: No statistically significant difference.

**Table 5:** Statistical analysis and outcome assessment prior and following PRP intraovarian infusion regarding the Menopausal pilot study.

**General Participants’ Characteristics**	**PRP Success (** ***n* = 13)**					**PRP Failure (** ***n* = 12)**					***p* Value**	
Participant age (years)	48.85 ± 1.57										<0.05	
Partner age (years)	49.15 ± 3.91										NS	
Duration of amenorrhea (months)	15.69 ± 1.75										NS	
Time to menstrual cycle recovery (days)	40.92 ± 7.57										NA	
**Outcome Measures Following PRP**	**Prior to Treatment**			**Follow-Up Month 1**			**Follow-Up Month 2**			**Follow-Up Month 3**		
	**PRP Success**	**PRP Failure**	***p* Value**	**PRP Success**	**PRP Failure**	***p* Value**	**PRP Success**	**PRP Failure**	***p* Value**	**PRP Success**	**PRP Failure**	***p* Value**
Menstrual cycle duration (days)	NA	NA	NA	35.62 ± 3.62	NA	NA	34.38 ± 4.52	NA	NA	32.77 ± 4.19	NA	NA
Menstruation duration (days)	NA	NA	NA	4.69 ± 0.95	NA	NA	4.69 ± 1.18	NA	NA	5.31 ± 1.65	NA	NA
FSH (IU/mL)	80.27 ± 5.03	81.15 ± 6.19	NS	43.63 ± 6.10	68.54 ± 7.26	<0.001	38.42 ± 2.50	65.40 ± 6.50	<0.001	30.55 ± 2.50	66.98 ± 8.12	<0.001
LH (IU/mL)	30.82 ± 3.78	30.18 ± 5.04	NS	22.62 ± 3.79	25.41 ± 4.23	NS	18.80 ± 3.80	24.65 ± 3.10	0.001	17.04 ± 3.08	26.60 ± 2.84	<0.001
AMH (ng/mL)	0.13 ± 0.03	0.11 ± 0.05	NS	0.32 ± 0.08	0.18 ± 0.08	<0.001	0.40 ± 0.13	0.24 ± 0.06	<0.001	0.61 ± 0.19	0.19 ± 0.04	<0.001
E_2_ (pg/mL)	14.01 ± 2.59	13.26 ± 2.30	NS	22.19 ± 5.63	16.31 ± 2.10	0.002	32.95 ± 2.73	15.29 ± 1.78	<0.001	41.53 ± 8.61	13.63 ± 2.30	<0.001
AFC	0	0	NS	1.31 ± 0.48	0	<0.001	1.77 ± 0.60	0	<0.001	2.38 ± 0.65	0	<0.001
**Spontaneous Pregnancies Recorded Following PRP**	**PRP Success**					**PRP Failure**					***p* Value**	
Number of Pregnancies achieved	1					0					NS	
Number of Live Births	1					0					NS	

NA: Not applicable; NS: No statistically significant difference.
